# Sex-dependent serotonergic signaling across development: molecular mechanisms shaping vulnerability to neurodevelopmental and mental disorders

**DOI:** 10.3389/fphar.2026.1800447

**Published:** 2026-04-23

**Authors:** Nicolás Palacios-Avendaño, Paulina S. Rojas, Matías Alarcón-Mardones, Jenny L. Fiedler

**Affiliations:** 1 Laboratory of Neuroplasticity and Neurogenetics, Department of Biochemistry and Molecular Biology, Faculty of Chemical and Pharmaceutical Sciences, Universidad de Chile, Santiago, Chile; 2 Faculty of Medicine, School of Pharmacy, Universidad Andres Bello, Santiago, Chile

**Keywords:** epitranscriptomics, mental disorders, neurodevelopment, serotonin, sex-dependent vulnerability, trophic effects

## Abstract

Psychiatric disorders and several neurodevelopmental conditions, including autism spectrum disorder, display marked sex biases in prevalence, symptom profiles, and treatment response. Converging evidence has positioned the serotonergic system as a key organizer of the brain during development and across the lifespan; however, the principles determining when serotonin acts permissively or instructively remains unresolved. In this Review, we critically examine interventional studies across sensitive developmental and adult windows to assess whether serotonergic signaling acts in a sex-dependent manner to shape circuit architecture and bias vulnerability to neurodevelopmental and psychiatric disorders. We argue that the consequences of serotonergic perturbation depend on developmental timing, biological sex, hormonal context, and circuit identity, rather than solely on serotonin levels. Finally, we discuss how staged interactions among serotonergic signaling, endocrine state, and circuit phenotypes—potentially modulated by RNA-centered regulatory processes—may offer a mechanistically plausible framework through which transient environmental challenges acquire lasting effects on neurodevelopmental and affective vulnerability.

## 1 Introduction

A central unresolved question in brain circuit formation is how early alterations in developmental processes give rise to sex-dependent trajectories of vulnerability to certain neurodevelopmental and psychiatric disorders, which may emerge later in life (Petrican et al., 2025). It is recognized that sexual brain dimorphism occurs early in development through variations in gonadal hormone exposure, particularly elevated testosterone levels during the fetal and early postnatal stages in males (Lombardo et al., 2012). These hormonal influences contribute to sex-dependent neurodevelopmental trajectories and continue to modulate circuit maturation and neuroplasticity across postnatal development and adulthood (Barth et al., 2015). However, hormonal mechanisms alone are insufficient to account for the complexity of sex differences in brain function and disease risk.

Experimental dissociation of chromosomal sex (XX or XY) from gonadal phenotype has demonstrated that neural cells can exhibit sex-specific phenotypic differences independently of circulating sex hormones, underscoring the contribution of additional regulatory layers beyond hormonal exposure (Carruth et al., 2002). Consistent with this view, although average sex differences in brain structure are well documented—often reflected as modest variations in regional volumes—such differences do not reliably predict behavioral, cognitive, or psychopathological outcomes (Peper et al., 2020). Instead, sex differences in brain development are more accurately reflected in patterns of interindividual variability, in the differential timing of vulnerability, and in the onset and phenotypic expression of disease. For instance, the early-onset neurodevelopmental conditions, such as autism spectrum disorder (ASD) and attention-deficit/hyperactivity disorder, are more prevalent in males (Werling and Geschwind, 2013; Loomes et al., 2017), whereas mood-related psychiatric disorders, including depression and anxiety, emerge later in life, showing a higher prevalence in females than males (Hankin, 2009; Breslau et al., 2017). These sex differences suggest the involvement of regulatory mechanisms at the level of gene expression that integrate biological sex, developmental timing, dependence on trophic molecules, and environmental factors—including stress exposure, pharmacological interventions, and nutrient availability—that may impact both circuit maturation and maintenance.

Importantly, neurotransmitters and neuromodulators play critical roles in shaping neural circuits during sensitive windows of mammalian central nervous system (CNS) development (Levitt et al., 1997; Herlenius and Lagercrantz, 2004). Perturbations in the timing, intensity, or spatial pattern of these signals during early life can durably alter cell proliferation and differentiation, receptor abundance, and circuit assembly, with consequences that may persist across the lifespan (Herlenius and Lagercrantz, 2004) and may underlie neurobehavioral and neurophysiological abnormalities. Among these signals, serotonin (5-hydroxytryptamine, 5-HT) is particularly relevant because it is present at very early embryonic stages and exerts developmentally important actions before assuming predominantly modulatory functions in the mature brain (Herlenius and Lagercrantz, 2004; Teissier et al., 2017; Deneris and Gaspar, 2018; Janet et al., 2023).

This Review is grounded in the premise that 5-HT availability exerts dual roles across the lifespan: during sensitive developmental windows, it acts instructively to guide neurocircuitry trajectories in a sex-dependent manner, whereas later it assumes a permissive role in sustaining established circuitries. We propose that sex differences in vulnerability to neurodevelopmental and psychiatric disorders may emerge from early perturbations in sex-dependent serotonergic mechanisms required to shape brain circuit architecture. By integrating developmental timing, circuit identity, and molecular regulation, we aim to advance a unifying framework that connects early disruptions in serotonergic organization to sex-biased susceptibility in both neurodevelopmental conditions and psychiatric disorders.

## 2 Serotonin as an early developmental signaling system

5-HT production is driven by a relatively small population of serotonergic neurons that reside in the dorsal and medial raphe nuclei of the midbrain, from which ascending fibers project broadly to cortical and subcortical regions (Puig and Gulledge, 2011; Kolk and Rakic, 2022; Ogelman et al., 2024; Ohmura and Nagayasu, 2025). This broad innervation highlights the role of the serotonergic system in orchestrating essential homeostatic processes—including sleep, thermoregulation, respiration, and food intake (Azmitia, 1999), while also modulating higher-order executive functions, such as mood regulation, cognition, learning, memory, and reward-related behaviors (Pourhamzeh et al., 2022).

A key determinant of these developmental actions is the tight regulation of 5-HT availability, governed by the spatiotemporal expression of two isoforms of tryptophan hydroxylase (TPH), the rate-limiting enzyme in the serotonin biosynthetic pathway (Walther and Bader, 2003). While TPH1 mediates peripheral and placental serotonin production, TPH2 provides the main source of brain serotonin (Walther and Bader, 2003; Walther et al., 2003). These two systems are functionally distinct, not only because different genes encode the two TPH isoenzymes, but also because 5-HT cannot cross the blood-brain barrier, making local regulation of 5-HT synthesis and availability essential for serotonergic signaling within the central nervous system (Walther and Bader, 2003).

Beyond its classical neurotransmitter and neuromodulator roles in the mature CNS, 5-HT also exerts early trophic functions that are critical for brain development, including the regulation of neurogenesis, neuronal migration, axonal guidance, and synaptic refinement before assuming predominantly modulatory functions in the mature brain (Herlenius and Lagercrantz, 2004; Teissier et al., 2017; Deneris and Gaspar, 2018; Janet et al., 2023). Importantly, these actions occur before intrinsic serotonergic projections have fully matured, requiring alternative sources of 5-HT during early embryogenesis. Consistent with this, constitutive *Pet-1* knockout (KO) mice, which exhibit a marked loss of serotonergic neurons, retain transient placental-derived 5-HT support to the embryonic forebrain between E10 and E15 (Bonnin et al., 2011), while offspring from *Tph1* KO pregnant mice display altered brain development, supporting a contribution of maternal and placental 5-HT to morphogenetic processes that precede the full establishment of fetal serotonergic neurons (Cote et al., 2007). Together, these findings indicate that early serotonergic signaling is developmentally instructive before raphe-derived fibers fully innervate the forebrain (Vitalis et al., 2007; Bonnin et al., 2011).

The placenta serves as a central regulator of the maternal–fetal interface, coordinating nutrient exchange, hormonal signaling, and immune adaptation to sustain fetal development (Khorami-Sarvestani et al., 2024). At this interface, serotonergic availability depends on tightly regulated transport and metabolic systems (Karahoda et al., 2020). Evidence from human term placenta suggests that altered expression of key proteins related to the serotonergic system—including increased TPH1/TPH2 and serotonin transporter (SERT) together with reduced monoamine oxidase A (MAO-A)—may be associated with early neurodevelopmental impairment in infants (Canul-Euan et al., 2025). Notably, 5-HT levels in maternal serum, umbilical cord serum, and placental tissue showed no differences, ruling out a predictive value of neurodevelopmental outcome in infants; supporting the view that the balance of placental serotonin signaling is relevant to early brain development (Canul-Euan et al., 2025). In addition, emerging evidence from constitutive SERT-deficient mice suggests that intracellular serotonergic handling at the maternal–fetal interface may influence development through direct epigenetic mechanisms (Chan et al., 2024). In this model, impaired placental 5-HT uptake was associated with reduced histone H3Q5 serotonylation—a serotonin-dependent chromatin modification—accompanied by a disruption of neurodevelopmental transcriptional programs in the brain (Chan et al., 2024). Importantly, at E12.5, brains from constitutive SERT KO mice—which at this stage do not express the enzymatic machinery required for 5-HT synthesis—showed serotonin levels comparable to those of wild-type animals. This observation suggests the existence of an alternative transport mechanism that sustains 5-HT transport to the brain during early development (Chan et al., 2024). However, the relative contribution of local placental serotonin synthesis versus maternally derived precursor supply in humans remains unresolved, highlighting a key gap in our understanding of how placental serotonergic regulation shapes fetal brain development and the later maturation of central serotonergic circuits (reviewed (Zhao and Bonnin, 2020; Peric et al., 2022))

## 3 Ontogeny of the serotonergic system: developmental timing, species differences, and early sex bias

**FIGURE 1:**
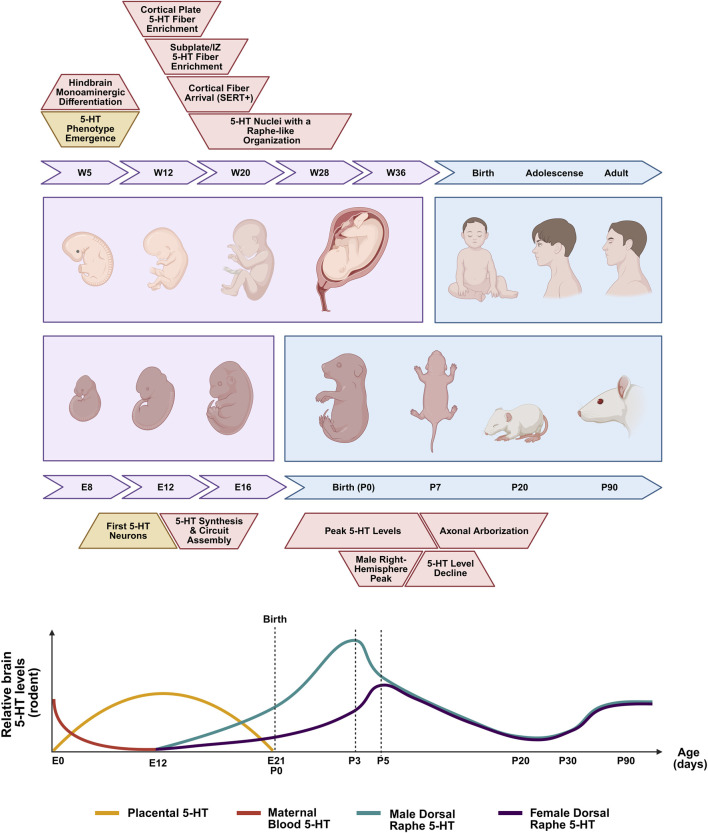
Ontogeny of the serotonergic system in humans and rats. Comparative timeline of structural development (*up*). The serotonergic system follows a precocious prenatal trajectory in humans. In contrast, rodent development is characterized by a protracted maturation that spans both pre- and postnatal stages. Dynamics of serotonin (5-HT) levels during rodent development (*down*). During the prenatal period, 5-HT levels are primarily sustained by maternal circulation (red line), which declines toward parturition, and placental 5-HT synthesis (yellow line). Postnatal maturation involves significant shifts in serotonergic signaling. Brain 5-HT levels exhibit sex-dependent temporal patterns: males show a 5-HT peak at postnatal day (P) 3 in the right hemisphere (teal line), while females exhibit a corresponding peak at P5 (purple line). Adapted from Suri et al. (2015). Created with BioRender.com.

Within the CNS, serotonergic neurons undergo early differentiation, playing a pivotal role in the maturation of other neurotransmitter systems (Daubert and Condron, 2010; Carvajal-Oliveros and Campusano, 2020) (Figure 1). In humans, immunohistochemical and neurochemical studies provide direct evidence that serotonergic neuronal phenotypes emerge early during brain development, with 5-HT-positive neurons detectable in the brainstem by gestational weeks 5–6, reflecting the early neurochemical differentiation of hindbrain monoaminergic lineages (Sundstrom et al., 1993). Analyses of monoaminergic markers further reveal a progressive and layer-specific distribution of serotonergic fibers within the developing cortex, with SERT-positive projections detected in the cortical anlage by approximately week 8, enriched in the subplate and intermediate zone around week 10, and extending into the cortical plate by around week 13 (Verney et al., 2002). By the mid-second trimester, serotonergic cell bodies display a recognizable raphe-like organization, accompanied by a pronounced lateral distribution within the brainstem, which is particularly prominent in humans (Takahashi et al., 1986). Thus, human fetal studies establish that serotonergic phenotypes emerge early and follow an anatomically ordered developmental sequence. However, because this literature is largely restricted to cross-sectional, marker-based fetal histology, it offers limited access to longitudinal sampling, causal perturbation, or sex-stratified circuit-level quantification, leaving key questions about sex-dependent and activity-dependent serotonergic maturation unresolved.

Rodent models, therefore, provide the primary experimental framework for defining temporal trajectories of serotonergic specification, axonal growth, and target-field refinement under controlled developmental staging. In rodents, serotonergic neurons first appear in the brainstem around embryonic day 10 (E10) (Takahashi et al., 1986), representing an early stage of embryonic neural development characterized by the initial specification of monoaminergic populations (Patel and Zhou, 2005). These neurons progressively establish widespread ascending and descending projections across the brain, forming highly collateralized axonal networks that innervate multiple target regions before the full maturation of their circuits (Lidov and Molliver, 1982; Deneris and Gaspar, 2018). The onset of 5-HT synthesis and release occurs around E13, temporally overlapping with neuronal differentiation and early circuit assembly (Patel and Zhou, 2005). Consistent with this early availability, serotonergic axons can release 5-HT before reaching their final targets, and this early signaling has been implicated in dendritic and axonal growth and arborization, likely in part through modulation of cytoskeletal dynamics (Speranza et al., 2015; Hamad et al., 2023). Together, these features position the serotonergic system to influence brain development from its earliest stages.

While embryonic development establishes the initial serotonergic framework, postnatal maturation is characterized by pronounced changes in 5-HT availability that coincide with a critical period of target innervation and circuit refinement. In rodents, brain 5-HT levels peak during the first postnatal week (Daws and Gould, 2011), with sex-dependent differences in timing, as concentrations peak at postnatal day (P) 3 in the right hemisphere of males and at P5 in females, suggesting an early sex bias in serotonergic availability (Connell et al., 2004) (Figure 1). From P5 to P14, both sexes show a gradual decline in 5-HT levels, whereas serotonergic axonal arborization and contact establishment with target regions continue until approximately P21 (Lidov and Molliver, 1982). Quantitative developmental mapping further reveals region-specific postnatal trajectories of serotonergic fiber maturation across cortical areas, the hippocampus—particularly the CA1 subfield—and the striatum, highlighting differential timing in synapse formation (Maddaloni et al., 2017). These temporal differences are consistent with early biochemical evidence that the serotonergic system also differs by sex at baseline. In rats, females show higher brain concentrations of 5-HT, its primary metabolite, 5-HIAA, and 5-HIAA/5-HT ratios than males, consistent with enhanced serotonergic turnover and presynaptic handling rather than simply increased synthesis (Carlsson and Carlsson, 1988). However, this pattern does not align with human findings, as positron emission tomography studies indicate lower rates of 5-HT synthesis in women than in men (Nishizawa et al., 1997; Barth et al., 2015). Thus, although sex-dependent differences in serotonergic biology are detectable from early developmental stages, the extent to which they shape the spatial organization, maturation, and subsequent functional output of serotonergic circuits—and how these processes relate to neurodevelopmental and neuropsychiatric disease vulnerability—remains unresolved.

## 4 Biochemical and behavioral consequences of altered serotonin availability across development and the lifespan

To dissect the role of 5-HT in early brain development, experimental manipulations of its availability—either reducing or enhancing serotonergic tone—demonstrate that alterations in serotonin signaling can exert long-lasting effects on brain organization and behavior (Adjimann et al., 2021), often in a sex-dependent manner (Table 1). A key distinction across these approaches is whether 5-HT availability is disrupted constitutively across the lifespan or transiently during specific developmental windows. Genetic models such as *Tph2* deletion produce lifelong reductions in brain serotonin, whereas SERT deletion elevates extracellular 5-HT by impairing reuptake. Pharmacological manipulations, including inhibition of TPH or blockade of SERT, allow transient reductions or elevations of serotonergic signaling during specific embryonic or postnatal periods. Collectively, these bidirectional models underscore developmental timing and biological sex as major organizing variables, while also highlighting the translational relevance of placental dysfunction, maternal stress, functional polymorphisms in serotonergic components, and developmental selective serotonin reuptake inhibitors (SSRI) exposure as plausible contexts of altered fetal and early-life 5-HT bioavailability (Hanswijk et al., 2020; Soto et al., 2024). In the following section, we will focus on evidence for sex differences in these genetic and pharmacological models, highlighting how serotonergic perturbations may differentially shape male and female neurodevelopmental trajectories (see also Table 1).

**TABLE 1:** Influence of biological sex on the serotonergic system during embryonic development and across the lifespan of the central nervous system (CNS) in preclinical models.

Type of serotonergic disturbance	Effect on 5-HT signaling	Species	Age of exposure	Sex	Effect on CNS	Behavioral effect	References
Genetic serotonergic deficiency: Constitutive *Tph2* KO (−/−)	Permanent brain 5-HT deficiency	Mice	Embryogenesis (lifelong)	♂, ♀ (Pooled)	Normal neuronal differentiation; ↑ 5-HT_1A/1B_ receptors in PFC, caudate-putamen, and septum	No sex differences in markers examined	Gutknecht et al. (2012)
		Rats	Adulthood	♂, ♀	↑ BDNF and mature BDNF in PFC; blunted stress-induced neuronal activation (Arc/cFos)	Blunted stress response; anticipated changes in behavioral stress challenges	Brivio et al. (2018)
				♂	Reorganization in response to sustained 5-HT loss	↓ Anxiety; heightened aggression; ↓ Social interaction; cognitive inflexibility	Meng et al. (2022) Alonso et al., 2023 Golebiowska et al., 2025
				♀		↑ Compulsivity; outcomes independent of ovarian hormones	Golebiowska et al. (2025)
Genetic serotonergic deficiency: Inducible *Tph2* KO	Adult-restricted ↓ 5-HT	Mice	Adulthood	♂	↑ Innervation in HIP; ↓ Innervation in the PVN of the thalamus	Defects rescued by 5-hydroxytryptophan administration	Pratelli et al. (2017)
Pharmacological 5-HT depletion: TPH inhibitor (PCPA)	Acute ↓ 5-HT	Mice	Embryonic development (GD8)	Not specified	Altered neuronal differentiation, growth, and dendritic/axonal arborization across cortical layers	Persistent alterations into adulthood (P80)	Khozhai and Otellin (2006)
	Transient ↓ 5-HT availability		Embryonic development (GD12.5–GD14.5)	♀	↓ *Tph2* mRNA in mPFC; ↓ D2 receptor expression; ↑ SERT levels (P22)	Compulsive-like behaviors (P21) and alcohol-induced anxiolytic effects (P28)	Fabio et al. (2022)
				♂, ♀	↓ SERT in NAc at adulthood (PD81)	↑ Non-social behaviors at weaning persisted until P78	Fabio et al. (2025)
	Transient postnatal ↓ 5-HT availability	Rats	Neonatal (P8–P16)	♂	↓ *Tph1/Tph2* mRNA in DRN; increased 5-HT_2C_ in amygdala (P60)	Bias in circuit maturation	Trujillo et al. (2021)
				♀	↑ 5-HT_2C_ in PFC at P17	↑ Anxiety-like behavior and ↓ self-grooming	
	Stage-specific depletion	Mice	Adolescence (P40–P42)	♀	Long-term programming of affective phenotypes	↓ Anxiety-like behavior persists into adulthood	Bellia et al. (2021)
Environmental/Maternal exposure: Maternal high-fat diet	Selective ↓ in placental and fetal forebrain 5-HT	Mice	Prenatal	♂	Inflammation-driven aberrant phagocytosis of serotonergic neurons	↓ Sucrose preference at 2 months of age	Ceasrine et al. (2022)
	Selective ↓ in placental 5-HT			♀	∅ in 5-HT levels at fetal forebrain	↓ Social interaction at the juvenile stage (P30)	
				♂, ♀	Not specified	∅ in anxiety-like behaviors at 3–4 months	
Pharmacological 5-HT elevation: Reuptake blockade (fluoxetine)	↑extracellular 5-HT	Rats	Prenatal gestational (GD0–P0)	♂	↓ Of many transcripts, especially in PFC and dHIP Anticipated maturation profile in the dentate gyrus	Anhedonia-like behavior in adulthood	Gallo et al. (2023), Gallo et al. (2026b)
			Postnatal lactational (P0–P21)	♀	↑ In target-gene expression, particularly in hippocampal subregions Delayed maturation profile in the dentate gyrus	Impaired novel object recognition; ↓ Anxious; ↑ Disinhibited exploratory profile	Gallo et al. (2023), Gallo et al. (2026b)
			Prenatal gestational (GD0–P0)	♂	Impaired endocrine and glucocorticoid receptor-mediated molecular response to acute stress	Latent vulnerability (no overt baseline behavior changes during adolescence)	Gallo et al. (2024)
			Perinatal windows (Pre, Post, or Combined)	♀	Subregion-specific hippocampal transcriptional signatures (neuroplasticity, microglia, mitochondrial, GABAergic markers)	Timing-dependent adult outcomes	Gallo et al. (2026a)
			Postnatal (P2–P21)	♂	Specific transcriptional programs and mitochondrial/dendritic remodeling in the mPFC.	↑ Anxiety- and despair-like behavior in adulthood	Ghai et al. (2026)
			Juvenile (P28–P48)	♂	Divergent mPFC transcriptional programs compared to postnatal treatment		Ghai et al. (2026)
Genetic alteration of serotonergic reuptake: Hyperfunctional SERT (Ala56 KI)	↑ 5-HT reuptake	Mouse	Embryogenesis (lifelong)	♂	↑ 5-HT clearance; 5-HT_1A/2A_ receptor hypersensitivity; altered dorsal raphe firing	↓ pup ultrasonic vocalizations; ↓ sociability; repetitive behavior	Veenstra-VanderWeele et al. (2012)
Genetic alteration of serotonergic reuptake: Hyperfunctional maternal SERT (Ala56)	↓ placental and embryonic forebrain 5-HT	Mouse	Prenatal (E14.5–E18.5)	♂, ♀ (Pooled)	Broadening of 5-ht-sensitive thalamocortical projections; altered fetal serotonergic environment	Not assessed	Muller et al. (2017)

Abbreviations: Serotonin (5-HT); tryptophan hydroxylase 1 and 2 (TPH1, TPH2); para-chlorophenylalanine (PCPA); brain-derived neurotrophic factor (BDNF); prefrontal cortex (PFC); medial prefrontal cortex (mPFC); hippocampus (HIP), dorsal hippocampus (dHIP); dorsal raphe nucleus (DRN); nucleus accumbens (NAc); paraventricular nucleus (PVN); gestational day (GD); embryonic day (E); postnatal day (P), as indicated in the original studies). ♀ = female; ♂ = male; ↑ = increased; ↓ = decreased; ∅ = no changes.

### 4.1 Sustained reductions in brain 5-HT: from constitutive deficiency to adult structural maintenance

The constitutive deletion of *Tph2* provides a genetic model of a permanent brain 5-HT deficiency that begins at embryogenesis. Importantly, *Tph2* ^*−/−*^ animals develop brain serotonergic neurons normally and display electrophysiological properties characteristic of the raphe nuclei, indicating that serotonin itself is not required for serotonergic neuronal differentiation (Gutknecht et al., 2012). The same study reported no sex differences in *Tph2* ^*−/−*^ mice across the markers examined; therefore, male and female data were pooled for subsequent analyses (Gutknecht et al., 2012). Importantly, this study described adaptive responses, including upregulation of 5-HT_1A_ and 5-HT_1B_ receptor protein levels in PFC, caudate-putamen, and septum (Gutknecht et al., 2012). In contrast, the CA1 area showed a more selective response, with upregulation confined to 5-HT_1A_ receptors (Gutknecht et al., 2012). Another study reported that adult male and female *Tph2* ^*−/−*^ rats exhibited a significant increase in brain-derived neurotrophic factor (BDNF) transcripts and mature BDNF peptide levels in the PFC compared to wild-type controls (Brivio et al., 2018). Although this study did not evaluate behavioral outcomes, it showed that acute stress-induced neuronal activation—quantified by increases in *Arc* and *cFos* transcripts in the PFC—was markedly blunted in both male and female *Tph2* ^*−/−*^ adult rats (Brivio et al., 2018). These suggest that a reduction in neuronal activity may be associated with reductions in norepinephrine content and the number of noradrenergic neurons in *Tph2* ^*−/−*^adult rats; neurons that are activated during the stress response (Gutknecht et al., 2012).

These studies indicate that female and male *Tph2* ^*−/−*^ animals undergo cross-monoaminergic reorganization in response to sustained 5-HT loss (Gutknecht et al., 2012), and the noradrenergic system—normally activated under stress (Privitera et al., 2024)—shows impaired responsiveness under acute stress exposure (Brivio et al., 2018) that may anticipate changes in behavioral responses under stress challenges. At the behavioral level, *Tph2* ^*−/−*^ male adult rats show reduced anxiety-like behavior and heightened aggression during social interactions (Meng et al., 2022), as well as diminished social interaction and communication together with increased repetitive behaviors (Alonso et al., 2023); phenotypes that resemble those observed in animal models of autism (Peralta et al., 2016). Importantly, a clear sex-specificity of autistic-like behavioral profiles has been described, i.e., males exhibit heightened aggression and cognitive inflexibility, whereas females display increased compulsivity; outcomes independent of fluctuations in ovarian hormones (Golebiowska et al., 2025).

While constitutive *Tph2* deficiency reveals the cumulative developmental and behavioral consequences of chronically reduced brain 5-HT, complementary evidence indicates that 5-HT is also required after development to preserve established serotonergic architecture. Using an inducible, adult-restricted *Tph2* conditional KO model, it was observed that depletion of brain 5-HT exclusively during adulthood leads to region-specific remodeling of serotonergic innervation (Pratelli et al., 2017). Selective reduction of 5-HT levels in the DRN and its axons produces an increase in innervation density within the hippocampus, and a concomitant reduction in the paraventricular nucleus of the thalamus (PVN), and these defects are rescued following reestablishment of brain 5-HT signaling via administration of the serotonin precursor 5-hydroxytryptophan (Pratelli et al., 2017). This study highlights that 5-HT is required throughout life to sustain serotonergic axonal growth in a region-dependent manner in adults.

Collectively, these studies suggest that sustained reductions in 5-HT elicit compensatory changes in receptor expression, accompanied by elevated levels of mature BDNF within cortical and basal ganglia circuits, while attenuating stress-induced neuronal activity. Although these molecular adaptations appear sex-independent, behavioral outcomes reveal autism-like phenotypes that differ between males and females. Because constitutive *Tph2* deletion imposes a permanent serotonin deficiency, it remains critical to determine whether these phenotypes reflect a disruption of 5-HT actions during specific developmental windows or result from cumulative effects of lifelong 5-HT loss. To address this issue, subsequent studies have used pharmacological approaches to transiently reduce serotonin synthesis or uptake during defined embryonic and postnatal periods (Table 1).

### 4.2 Transient developmental reductions in 5-HT availability

During early gestation, as we discuss in Section 1, the placenta acts as the primary interface regulating fetal serotonergic exposure before intrinsic serotonergic projections fully mature. Although maternal 5-HT itself rarely crosses the placenta, its precursor tryptophan does, providing substrate for placental 5-HT synthesis. Under conditions such as maternal stress, altered precursor availability may therefore reshape placental and fetal serotonergic signaling, offering a plausible route by which gestational physiology influences later neurodevelopmental and behavioral outcomes (Rakers et al., 2017).

One widely used pharmacological approach to induce an acute depletion of 5-HT levels is the administration of p-chlorophenylalanine (PCPA), an inhibitor of TPH. Several studies in rodents have manipulated 5-HT production during early or late gestational windows—developmental periods in which specific cortical and limbic regions, particularly the medial prefrontal cortex (mPFC), remain partially dependent on non-neuronal sources of serotonin prior to the full maturation of ascending raphe projections. Additionally, some studies conducted longitudinal analyses of prenatal PCPA administration to detect persistent, circuit- and sex-specific phenotypes. For instance, the single PCPA administration at gestational day (GD) 8 alters neuronal differentiation, growth, and dendritic and axonal arborization across cortical layers in P1-P10, and this effect persists into adulthood (P80) (Khozhai and Otellin, 2006). This alteration occurs in the absence of evident changes in serotonergic circuitries, suggesting an important role of extrinsic serotonin during early gestational development (Khozhai and Otellin, 2006). PCPA administration during mid-gestation (GD12.5–14.5) also induces long-lasting consequences at weaning (P22), evidenced by reduced *Tph2* mRNA expression in the mPFC of both sexes, but by low dopamine 2 receptor (D2R) expression and high SERT levels observed only in females (Fabio et al., 2022). Authors suggested that reduced expression of the D2R may influence stress responsiveness in female mice; however, this possibility remains unexplored (Fabio et al., 2022). On the other hand, the combined effect of reduced 5-HT synthesis and potentially enhanced 5-HT uptake in the female mPFC suggests diminished serotonin availability at weaning (Fabio et al., 2022), anticipating sex-specific alterations in behavioral outcomes. Contrary to this expectation, early PCPA administration (GD12.5–14.5) was associated with increased compulsive-like behaviors at P21 and alcohol-induced anxiolytic effects during early adolescence (P28) in both sexes, suggesting selective vulnerability of specific behavioral domains rather than a global dysfunction (Fabio et al., 2022). Notably, this study did not observe stereotyped behaviors or alterations in social preference and social novelty at weaning in either sex (Fabio et al., 2022), in contrast to the findings in Tph^−/−^ animals (Golebiowska et al., 2025), suggesting that 5-HT is required not only within specific temporal windows during fetal brain development but also across the lifespan.

Notably, the PCPA administration from GD12.5 to GD14.5 triggers an increment in non-social behaviors observed at weaning that persist until the last observation conducted at P78, a reduction in SERT in nucleus accumbens (NAc) at adulthood (P81); although the sex factor was collapsed in this analysis (Fabio et al., 2025). On the other hand, SERT immunoreactivity in the NAc was reduced in both males and females, suggesting that variations in 5-HT levels may be linked with impairments in social behavior observed at different developmental stages (Fabio et al., 2025). The authors suggested divergent maturation trajectories of cortico-limbic serotonergic circuits. Together, these findings support a model in which transient reductions in 5-HT availability during late gestation act in an organizational manner, constraining the maturation of social and reward-related circuits across development (Fabio et al., 2025).

Using neonatal administration of PCPA between postnatal days P8 and P16, it was demonstrated that transient postnatal 5-HT depletion induces persistent, sex-specific alterations in serotonergic gene expression and behavior (Trujillo et al., 2021). In males, PCPA treatment resulted in long-lasting changes that led to reduced expression of *Tph1* and *Tph2* within the dorsal raphe nucleus (DRN), whereas females displayed more pronounced behavioral alterations, including increased anxiety-like behavior and reduced self-grooming, highlighting sex differences in vulnerability to postnatal serotonergic disruption (Trujillo et al., 2021). Follow-up studies further revealed region-specific and sex-dependent changes in serotonergic receptor expression, including increased 5-HT_2C_ receptor levels in the PFC of females at P17 and in the amygdala of adult males at P60, reinforcing the notion that transient serotonergic perturbations during early life bias circuit maturation in a sex- and region-dependent manner (Trujillo et al., 2021). Importantly, developmental timing further constrains the direction and persistence of behavioral outcomes. Neonatal 5-HT depletion during adolescence (P40–42), but not during early infancy, reduced anxiety-like behavior, an effect that persisted into adulthood in females, supporting the existence of stage-specific windows in which serotonergic disruption differentially programs long-term affective phenotypes (Bellia et al., 2021).

To sum up, pharmacological and genetic approaches converge on the conclusion that 5-HT plays permissive and instructive roles during early brain development, such that even transient embryonic reductions in serotonergic availability can have lasting consequences (Table 1). Importantly, environmental exposures may similarly impact brain development by disrupting 5-HT homeostasis. In line with this, in a mouse model, maternal exposure to a high-fat diet prior to mating and throughout pregnancy selectively reduces 5-HT levels in the placenta and fetal forebrain of male offspring, while females remain unaffected, despite stable maternal serotonin levels. At the juvenile stage (P30), females exhibit reduced social interaction, whereas males do not; conversely, at 2 months of age, males show decreased sucrose preference, while females remain unaffected (Ceasrine et al., 2022). Furthermore, neither males nor females show anxiety-like behaviors at 3–4 months. Mechanistically, this model implicates inflammation-driven aberrant phagocytosis of male serotonergic neurons as a potential link between prenatal environmental challenge, sex bias, and persistent reductions in brain 5-HT levels (Ceasrine et al., 2022).

The long-lasting morphological alterations induced by PCPA were not accompanied by changes in cortical proBDNF levels during postnatal stages (P3-P21) (Vitalis et al., 2007); changes that contrasted with the *Tph2* ^*−/−*^ model, which observed cortical mBDNF in adult animals (Brivio et al., 2018). Mechanistically, the impairment in neuronal arborization is consistent with the role of 5-HT in activating 5-HT_7_ receptors, which promote cytoskeletal remodeling and axonal elongation in cortical neurons (Speranza et al., 2015).

### 4.3 Genetics and antidepressant exposure influence 5-HT transporter-activity: impact on brain development

A complementary line of evidence comes from conditions that reduce SERT function, either genetically or pharmacologically, thereby increasing extracellular 5-HT. In humans, functional variation in the 5-HT transporter-linked polymorphic region (5-HTTLPR) has been widely interpreted as evidence that altered reuptake biology can shape individual differences in serotonergic responsivity, although most such data derive from adult clinical or behavioral contexts rather than developmental models (Gressier et al., 2014; Muller et al., 2017; Martinez et al., 2020) are coding variation in SLC6A4, the gene encoding SERT in humans, further suggests that altered transporter function may contribute to neuropsychiatric vulnerability through mechanisms extending beyond canonical pharmacological blockade (Murphy and Moya, 2011).

In ASD families with linkage to chromosome 17q, the rare variants have been reported, the most frequent being Ala56, associated with rigid-compulsive traits and sensory hypersensitivity (Stilley and Blakely, 2021). More developmentally informative are transgenic studies of the ASD-associated Ala56 variant. In this model, male Ala56 knock-in mice show altered central 5-HT signaling and ASD-relevant phenotypes (Veenstra-VanderWeele et al., 2012), while maternal Ala56 expression disrupts fetal 5-HT system development, broadens 5-HT-sensitive thalamocortical projections, and is associated with reduced embryonic forebrain 5-HT at E14.5 in embryos from homozygous dams (Muller et al., 2017). Together, these findings support the view that altered SERT activity may influence neurodevelopment not only through direct changes in reuptake, but also indirectly through maternal–placental effects on the prenatal environment, a mechanism that remains unresolved (Muller et al., 2017; Stilley and Blakely, 2021).

Developmental SERT blockade with the antidepressant fluoxetine (FLX), by contrast, provides a more direct framework for examining how increased extracellular 5-HT during sensitive periods affects long-term circuit organization. FLX is particularly relevant because SSRIs are prescribed during pregnancy, breastfeeding, childhood, and adolescence, thereby exposing the developing brain to compounds that cross the placental barrier and are present in breast milk (Ghai et al., 2026). Rodent postnatal exposure paradigms may therefore provide a useful approximation of late gestational or third trimester (Figure 1) 5-HT perturbation in humans.

In rats, perinatal FLX exposure during gestation (GD0–birth) or early postnatal life (P0–P21) produced sex- and age-dependent adult outcomes, with rodent postnatal exposure likely modeling aspects of late gestational or third-trimester serotonergic perturbation in humans. For instance, prenatal FLX exposure preferentially triggers anhedonia-like behavior in males, suggesting selective vulnerability of reward-related cortico-limbic circuits (Hoflich et al., 2019); whereas postnatal FLX exposure preferentially impaired novel object recognition in females, consistent with altered maturation of hippocampal-dependent recognition-memory circuits, and also shifted elevated-plus-maze behavior toward a less anxious or more disinhibited exploratory profile (Gallo et al., 2023). These behavioral differences were later linked to distinct alterations in markers regulating sensitive-period dynamics, in which prenatal FLX in males was consistent with an anticipated maturation profile of dentate gyrus in hippocampus, whereas postnatal FLX in females was consistent with a delayed one (Gallo et al., 2026b). Moreover, prenatal FLX (GD0–birth) exposure did not overtly alter baseline behavior during adolescence in males, but it did impair the endocrine and glucocorticoid receptor–mediated molecular response to acute stress, suggesting that early serotonergic perturbation can establish a latent vulnerability state before an adult phenotype becomes fully manifest (Gallo et al., 2024).

Importantly, these studies also show that the consequences of developmental FLX exposure are strongly timing-dependent and extend beyond behavior to long-lasting circuit-level signatures in the hippocampus and mPFC (Gallo et al., 2026a; Ghai et al., 2026). In female rats, prenatal, postnatal, and combined perinatal FLX exposure left subregion-specific hippocampal transcriptional signatures in adulthood, differentially affecting neuroplasticity-, microglial-, perineuronal net-, mitochondrial-, GABAergic-, and autophagy-related markers across dorsal and ventral hippocampus (Gallo et al., 2026a). Likewise, direct comparison of postnatal (P2–P21) versus juvenile (P28–P48) FLX exposure can produce opposing adult outcomes, as postnatal treatment increased anxiety- and despair-like behavior in males, whereas juvenile treatment decreased both, in parallel with minimally overlapping transcriptional signature and divergent mitochondrial and dendritic remodeling in the mPFC (Gallo et al., 2026b). Taken together, these findings argue against a simple or unidirectional model of sex bias and instead support the view that biological sex, developmental window, and circuit identity jointly determine how early elevations in serotonergic tone are later expressed as anhedonic, cognitive, stress-reactive, or anxiety-related phenotypes.

## 5 Serotonergic receptors as modulators of neuroplasticity

The evidence described above establishes that 5-HT perturbations exert long-lasting, stage-dependent effects, underscoring the need to identify which 5-HT receptors and downstream pathways influence circuit- and behavioral outcomes.

### 5.1 Receptor diversity as a source of plastic specificity

Despite the relatively small number of serotonin-producing neurons in the mammalian CNS, their extensive and highly collateralized projections confer broad modulatory influence across cognition, emotion, and stress responsiveness (Pourhamzeh et al., 2022). Serotonergic signaling operates not only through classical synaptic transmission but also via volume transmission, enabling engagement with widely extra-synaptic distributed receptor populations (Gianni and Pasqualetti, 2023). Consequently, the functional impact of 5-HT depends critically on receptor subtype distribution, signaling properties, and stimulus-dependent regulation of receptor abundance.

**FIGURE 2:**
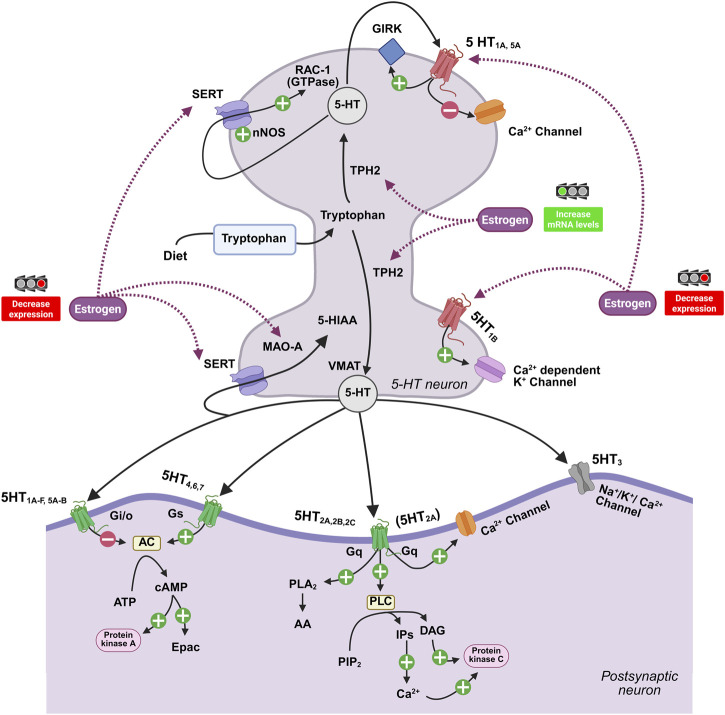
Estrogenic modulation of 5-HT synapse dynamics. The serotonergic receptor landscape comprises seven distinct families (5-HT_1-7_ receptors). Most function as G protein-coupled receptors (GPCRs), with the notable exception of the ionotropic 5-HT_3_ receptor, which mediates rapid excitatory neurotransmission. Estrogens exert multiple influences on this system in the presynaptic region. Estrogens modulate the expression of the serotonin transporter (SERT), the monoamine oxidase (MAO), and the rate-limiting enzyme tryptophan hydroxylase 2 (TPH2), as well as the sensitivity of 5-HT_1A/1B_ autoreceptors. This hormonal regulation is critical for maintaining homeostatic balance in circuits governing mood, cognition, and neurodevelopment. Adapted from Paredes et al. (2019). Created with BioRender.com.

Serotonergic receptors comprise seven families encoded by at least fifteen genes, most of which are G protein–coupled receptors, apart from the ionotropic 5-HT_3_ receptor (Albert and Tiberi, 2001; Mattson et al., 2004; Pourhamzeh et al., 2022) (Figure 2; Table 2). These subtypes exhibit distinct temporal expression patterns and regional enrichment, conferring functional specificity to serotonergic modulation (Tan et al., 2022). Additional complexity arises from alternative splicing, RNA editing (see below), and receptor heterocomplex formation, which diversify signaling outcomes (Rojas and Fiedler, 2016; Warhaftig et al., 2021; Pourhamzeh et al., 2022; Bijata et al., 2024). Thus, variations in extracellular 5-HT availability do not produce uniform effects but are filtered through receptor composition and local regulatory context, particularly during periods of heightened plasticity.

**TABLE 2:** Serotonin receptor subtypes in the human brain: signaling mechanisms, distribution, putative functions, and associated pathologies.

5-HT receptor subtype	Transduction system	Human brain regions	Putative function	Related clinical relevance
5-HT_1A_	GPCR (Gi/o → ↓cAMP; GIRK activation)	DRN, Hip, Hyp, Amy, Ctx, CPu, FCtx	Serotonergic neurotransmission; thermoregulation; feeding; stress; pain; mood; emotion; cognition; learning; memory	Anxiety/Depression; neurodegenerative disorders; schizophrenia
5-HT_1B_	GPCR (Gi/o → ↓cAMP)	SN/VTA, NAc, CPu, VP, Ctx	Serotonergic neurotransmission; mood; feeding	Anxiety/Depression; migraine
5-HT_1D_	GPCR (Gi/o → ↓cAMP)	CPu, VP, FCtx	Serotonergic neurotransmission; mood; feeding	Anxiety/Depression; migraine
5-HT_1E_	GPCR (Gi/o → ↓cAMP)	CPu, Hip, Ctx	Unknown	Unknown
5-HT_1F_	GPCR (Gi/o → ↓cAMP)	Cb, Hip, Ctx	Mood; emotion	Migraine
5-HT_2A_	GPCR (Gq/11 → PLC)	DVC, Thal, CPu, Ctx, FCtx	Mood; respiratory control; feeding; nociception	Depression; Tourette’s syndrome; Alzheimer’s disease; anorexia; bulimia; drug abuse; pain
5-HT_2B_	GPCR (Gq/11 → PLC)	Cb?, LS?, Hip?, Ctx?	Brain development?	Feeding?; drug abuse; anxiety?
5-HT_2C_	GPCR (Gq/11 → PLC)	ChP, Cb, DRN, SN, Hyp, Amy, Hip, CPu, NAc, Ctx	Mood; impulsivity; feeding; locomotor activity	Anxiety/depression; schizophrenia; drug abuse; obesity
5-HT_3_	Ligand-gated ion channel (Na^+^, K^+^, Ca^2+^ conductance)	DVC, Hip, Amy, CPu	Vomiting reflex; mood	Nausea; anxiety/Depression
5-HT_4_	GPCR (Gα13, Gs → ↑cAMP)	Hyp, Hip, NAc, CPu	Feeding; reward; cognition	Anorexia; drug abuse; Alzheimer’s disease
5-HT_5A_	GPCR (Gi/o → ↓cAMP)	Cb, Hyp, Thal, Hip, Ctx	Circadian rhythm; sleep; mood; cognition	Schizophrenia?; anxiety/Depression?
5-HT_5B_	-	Not expressed in humans	-	-
5-HT_6_	GPCR (Gs → ↑cAMP)	Hip, CPu, Ctx, OT	Cognition; learning; memory; feeding	Alzheimer’s disease; dementia; obesity
5-HT_7_	GPCR (G12, Gs → ↑cAMP)	DRN, Hyp, Thal, Hip, Amy, Ctx	Mood; sleep; cognition; pain; migraine	Anxiety/Depression; schizophrenia

Abbreviations: DRN: dorsal raphe nucleus; Hip: hippocampus; Hyp: hypothalamus; Amy: amygdala; Ctx: cortex; FCtx: frontal cortex; CPu: caudate–putamen; NAc: nucleus accumbens; VP: ventral pallidum; SN: substantia nigra; VTA: ventral tegmental area; Thal: thalamus; Cb: cerebellum; DVC: dorsal vagal complex; LS: lateral septum; ChP: choroid plexus; OT: olfactory tubercle. Adapted from Charnay, Dutton and De Deurwaerdère (Charnay and Leger, 2010; Dutton and Barnes, 2008; De Deurwaerdere and Di Giovanni, 2017). Question marks indicate tentative or indirect evidence derived from limited human data or extrapolated from animal studies. ↑ = increased; ↓ = decreased.

Recent single-cell transcriptomic mapping further reveals that serotonergic receptor expression is organized in highly cell-type–specific and combinatorial patterns, generating micro-architectures of 5-HT responsiveness within and across brain regions (De Filippo and Schmitz, 2024). This structural complexity implies that even subtle regulatory shifts in receptor transcript abundance, isoform composition, or RNA processing could disproportionately reshape serotonergic signaling output. In this context, sex differences may not necessarily arise from global variations in 5-HT levels, but rather from differential regulation of receptor-expression landscapes at the transcriptomic level.

### 5.2 Developmental and stress-dependent receptor control of plasticity

Beyond structural diversity, receptor heterogeneity carries functional consequences: distinct 5-HT receptor families engage divergent intracellular programs that regulate synaptic strength, dendritic remodeling, and gene transcription (Higa et al., 2024). In this framework, shifts in receptor composition—driven by developmental, stress-related, or sex-dependent factors—may reshape serotonergic signaling by engaging different plasticity mechanisms within specific circuits.

Evidence from developmental studies supports this view. In rodents, 5-HT_1A_ receptor mRNA is detectable in the fetal brain as early as E12, reaching peak levels around mid-gestation (E15) and declining toward birth (E20), with early expression reported in the raphe nuclei and hippocampus and transient postnatal detection in motor neurons and the cerebellum (Hillion et al., 1993; Miquel et al., 1994). Additionally, during the early postnatal period, disruption of serotonergic output reduces excitatory synapse density and synaptic strength in the developing PFC, whereas increased 5-HT availability promotes long-term potentiation and dendritic spine formation through 5-HT_2A_ and 5-HT_7_ receptors in a glutamate-independent manner (Ogelman et al., 2024). Together, these findings indicate that receptor-specific serotonergic signaling contributes directly to early circuit assembly and synaptic maturation.

Serotonergic receptor regulation also remains highly sensitive to environmental challenges later in life. Activation of the 5-HT_1A_ receptor has been shown to increase progenitor proliferation in adult neurogenic niches such as the hippocampus and subventricular zone (Yan et al., 1997; Brezun and Daszuta, 1999), processes that are strongly modulated by stress exposure and glucocorticoid signaling (Mirescu and Gould, 2006). In line with this, repeated restraint stress decreases hippocampal 5-HT_1A_ receptor mRNA levels in females but not in males (Goel et al., 2022), highlighting the emergence of sex-specific receptor regulation under stress conditions.

Recent work further illustrates how receptor landscapes can be dynamically reconfigured in a sex-dependent manner. In hippocampal neural stem cells (NSC), females predominantly express 5-HT_1A_ receptors, whereas males show higher expression of 5-HT_7_ receptors. Under chronic restraint stress (CRS) or selective 5-HT_1A_ receptor deletion, females exhibit compensatory upregulation of 5-HT_7_ receptors in both conditions, whereas males display a stress-dependent downregulation of 5-HT_7_ receptors (Luo et al., 2024). This receptor reconfiguration is accompanied by a reduction of the NSC pool in females but not in males, suggesting that stress-induced receptor remodeling may differentially affect cellular plasticity across sexes, although behavioral consequences remain unresolved.

Converging evidence indicates that chronic stress also induces region- and sex-specific adaptations across additional serotonergic receptor families. For instance, stress enhances 5-HT_1A_ autoreceptor signaling in the DRN of male rats, while females exhibit increased 5-HT_1A_ responsiveness in the hippocampus following stress exposure (Philippe et al., 2022). In addition, in other study using a rat models of restraint stress, 5-HT_1B_ expression decreases in both the PFC and hippocampus in both sexes, whereas the amygdala exhibits a sex-divergent profile, with increased 5-HT_1B_ expression in males but decreased expression in females; these receptor changes co-occur with pronounced anxiety-like behavior and sustained endocrine responses in females (Siddiqui et al., 2025). These findings are translationally relevant, as downregulation of inhibitory serotonergic receptors—including 5-HT_1B_—has been proposed as a mechanism contributing to the delayed therapeutic effects of SSRIs in major depressive disorder (MDD), positioning 5-HT_1B_ as a potential antidepressant target (Tiger et al., 2018). Together, these observations suggest that stress-induced remodeling of 5-HT_1B_ signaling may contribute to sex-dependent vulnerability to mood-related phenotypes rather than reflecting a uniform adaptive response of the serotonergic system.

Similar region- and sex-dependent regulatory patterns extend to other inhibitory serotonergic receptors. In rats exposed to CRS, 5-HT_5B_ expression decreases in the PFC but increases in the hippocampus in both sexes, whereas 5-HT_5A_ expression is selectively upregulated in the amygdala, with a greater magnitude in females than in males (Siddiqui et al., 2025). Although females in this model display stronger anxiety-like and endocrine stress responses, direct causal links between 5-HT_5A/5B_ receptor regulation and behavioral outcomes remain unresolved. Collectively, these findings indicate that chronic stress reshapes serotonergic signaling through region-specific and sex-biased remodeling of inhibitory receptor landscapes.

## 6 Hormonal context as a modulator of serotonergic transmission

### 6.1 Hormonal tuning of serotonergic tone

Across experimental models, estrogen has been shown to increase brain serotonin levels and to regulate key enzymatic steps involved in serotonin synthesis and degradation, including TPH and MAO-A, although the direction and magnitude of these effects vary across brain regions and exposure paradigms (Epperson et al., 2012; Barth et al., 2015) (Figure 2). In ovariectomized rats, estradiol replacement selectively increased TPH2 mRNA in discrete DRN/MRN subregions, whereas combined estradiol–progesterone treatment abolished this induction, indicating hormone- and context-dependent control of serotonergic synthetic capacity (Hiroi et al., 2006). Notably, TPH2 expression correlated with anxiety-related measures in a subregion-specific manner, underscoring that “more synthesis” does not translate into a unitary behavioral direction across raphe modules (Hiroi et al., 2006). In line with this, in a recent study using an ovariectomy mouse model, in which disruption of estrogen receptor (ER) signaling—particularly reduced ERβ and altered ERα expression across multiple brain regions—is associated with impairments in cognition and the emergence of depressive-like phenotypes, highlighting the vulnerability that follows loss of ovarian hormones (Baek et al., 2024). More broadly, recent evidence suggests that sex-biased vulnerability may arise not only from differences in hormone levels but also from sex-divergent receptor engagement under comparable local endocrine states. In the hippocampus, high local estradiol was associated with stress susceptibility in both males and proestrus females, yet this effect was mediated by ERα in males and ERβ in females, highlighting that similar phenotypic outcomes can emerge through sex-specific molecular routes (Hokenson et al., 2026).

### 6.2 Hormonal modulation of serotonergic receptor function

Estrogen differentially regulates serotonergic receptor systems rather than uniformly enhancing serotonergic signaling. It increases 5-HT_2A_ receptor mRNA in cortical regions implicated in cognition and mood (Sumner and Fink, 1998), downregulates inhibitory autoreceptors such as 5-HT_1B_ in the DRN (Hiroi and Neumaier, 2009), and can suppress 5-HT_1A_ receptor mRNA expression and binding in a treatment-duration–dependent manner (Pecins-Thompson et al., 1998; Osterlund and Hurd, 2001). Progesterone exerts partially overlapping effects, including reduced 5-HT_1A_ receptor expression in rodents and combined estrogen–progesterone–dependent suppression of 5-HT_2C_ receptor expression in non-human primates (Henderson and Bethea, 2008). Together, these findings indicate that hormonal state reshapes receptor landscapes across brain regions, providing a mechanistic substrate through which sex and reproductive state may bias serotonin-dependent plasticity.

This hormone sensitivity extends to pharmacological responsiveness. Sex steroids modulate SSRI effects (Benmansour et al., 2016), and hormonal status interacts with genetic variation in serotonin transporter regulation to shape antidepressant outcomes in both primate models and humans (Michopoulos et al., 2011; Gressier et al., 2014). Converging rodent and human evidence further implicates the 5-HT_4_ receptor as a hormone-sensitive node linking serotonergic signaling to mood and cognition: its activation is required for full fluoxetine efficacy in rodents (Lucas et al., 2007; Mendez-David et al., 2014), whereas reduced receptor binding in humans is associated with MDD and memory dysfunction (Kohler-Forsberg et al., 2023). Finally, circulating sex hormones correlate with this receptor binding in healthy individuals—negatively with testosterone and positively with estradiol in men—relationships that appear disrupted under depressive states, further supporting endocrine context as a modulator of receptor-level serotonergic phenotypes in humans (Aarestrup et al., 2025).

## 7 RNA-level regulation in the maintenance and adjustment of serotonergic states

A central challenge in linking altered serotonergic signaling to neurodevelopmental and psychiatric disease vulnerability is understanding how early disruptions in 5-HT function can induce lasting effects. Notably, mRNA abundance alone often fails to predict receptor availability, transporter expression, or downstream signaling capacity, suggesting that intermediate RNA-level regulatory layers may define the efficiency of serotonergic signaling—here conceptualized as the integrated configuration of receptor composition, transporter availability, and downstream signaling properties that determine functional serotonergic tone—not captured by conventional gene expression analyses.

Epigenomic regulation has been extensively investigated as a mechanism linking environmental exposures to persistent variation in serotonergic signaling (Yuan et al., 2023). Across animal models and human studies, DNA methylation, histone modifications at serotonergic loci—including SERT and selected 5-HT receptor genes—have been associated with stress responsivity and affective traits (Fass et al., 2014; Wu T. et al., 2023; Javelle et al., 2025). However, while chromatin-level control provides a stable regulatory backdrop, it does not fully account for RNA-level processes that shape transcript processing, isoform diversity, translation efficiency, and protein output in serotonergic systems.

### 7.1 Transcript stability and translational accessibility in serotonergic signaling

Post-transcriptional mechanisms that regulate transcript stability, 3′UTR usage, and translational accessibility represent an RNA-level layer of control that can modulate serotonergic tone, contributing to the uncoupling between the transcriptome and proteome. One of the earliest examples in this context is the SERT polyadenylation polymorphism, which promotes longer 3′UTR isoforms associated with reduced translational efficiency and altered microRNA binding, resulting in persistent modulation of serotonergic output and stress-related memory phenotypes (Hartley et al., 2012; Yoon et al., 2013). Beyond alternative polyadenylation, stress-responsive microRNAs provide an additional post-transcriptional interface between environmental exposure and serotonergic circuitry. For example, upregulation of miR-17-5p in the ventral hippocampus of adult male rats remodels synaptic and serotonergic gene networks—including pathways involving the 5-HT_2C_ receptor—and produces antidepressant-like effects under stress (Olave et al., 2026), illustrating how microRNA-mediated translational control can recalibrate serotonergic tone in a context-dependent manner.

Despite their mechanistic relevance, alternative polyadenylation and microRNA-mediated regulation have rarely been examined in sex-stratified designs, limiting conclusions about their contribution to sex-biased serotonergic vulnerability. Together, these RNA-level mechanisms regulate transcript stability and accessibility without altering nucleotide chemistry, raising the question of how RNA modifications may further diversify serotonergic regulation.

### 7.2 RNA chemical modifications as dynamic modulators of serotonergic plasticity

Chemical modifications on RNA nucleotides have given rise to the field of epitranscriptomics, a post-transcriptional regulatory layer that expands the functional potential of the transcriptome (Sun et al., 2023). Through transcript-, site- and context-specific regulation, epitranscriptomic mechanisms can modulate RNA fate and protein output with high spatial and temporal precision, providing a flexible interface between cellular state and gene expression programs (Madugalle et al., 2020). Because RNA chemical marks can be installed and removed in a temporally restricted manner, they offer a mechanism through which developmental serotonergic perturbations may influence protein output during sensitive windows, potentially biasing subsequent circuit maturation. In particular, receptor composition, transporter availability, and synthetic capacity are all features that can be tuned post-transcriptionally and, in a compartment- and state-dependent manner—features that parallel the sex- and stress-related phenotypes summarized in previous Sections 4, 5.

In the brain, adenosine-to-inosine (A-to-I) RNA editing and N^6^-methyladenosine (m^6^A) have emerged as the best-characterized epitranscriptomic marks on neuronal mRNAs, where they shape synaptic function from early stages of development, as well as stress responsiveness, and plasticity through distinct but complementary molecular mechanisms (Angelova et al., 2018; Flamand and Meyer, 2019; Barbon and Magri, 2020). Current evidence positions A-to-I editing and m^6^A not as definitive master regulators, but as plausible molecular substrates linking transient serotonergic perturbation to persistent circuit consequences.

### 7.3 RNA editing: an activity- and stress-sensitive regulator of serotonergic function

A-to-I RNA editing dynamically modifies the coding potential and regulatory properties of neuronal transcripts without altering the underlying DNA sequence (Tariq and Jantsch, 2012). Catalyzed by adenosine deaminases acting on RNA (ADARs), which are highly expressed in the central nervous system (Zhai et al., 2022; Liu et al., 2024), A-to-I editing expands proteomic diversity in a manner that is sensitive to neuronal activity and environmental context. In neurons, editing is enriched in transcripts encoding neurotransmitter receptors, ion channels, and synaptic regulators, where it can alter amino acid identity, splicing patterns, or RNA stability, thereby directly shaping protein function and excitability (Li et al., 2014; Terajima et al., 2018).

Importantly, RNA editing capacity itself follows a regulated developmental trajectory. Across mammalian neural tissues, ADAR2 expression and editing activity increase from embryonic to postnatal stages. In human embryonic stem cells, global A-to-I editing increases during neuronal differentiation, in parallel with ADAR2 upregulation (Shtrichman et al., 2012). Similarly, in porcine and rodent brains, ADAR2 mRNA levels increase during embryogenesis and early postnatal maturation, accompanied by progressive editing of synaptic transcripts (Hang et al., 2008; Jacobs et al., 2009; Veno et al., 2012). Notably, this regulation is isoform-specific: whereas ADAR1 expression remains relatively stable, ADAR2 exhibits robust postnatal upregulation together with the emergence of self-editing activity (Hang et al., 2008). These convergent findings indicate that RNA editing capacity is developmentally configured rather than constitutive, potentially defining temporally restricted windows during which diversification of serotonergic signaling components may interact with circuit maturation. Beyond its ontogenetic modulation, ADAR activity remains responsive to neuronal activation and stress exposure, evidencing a molecular interface through which environmental challenges may reshape serotonergic signaling (Terajima et al., 2018; Zhai et al., 2022; Wang et al., 2023). Within the serotonergic system, RNA editing is one of the few epitranscriptomic mechanisms for which direct effects on 5-HT levels and receptor-specific functional consequences on signaling and behavior have been experimentally demonstrated.

At the level of 5-HT synthesis, human *TPH2* pre-mRNA undergoes alternative splicing and extensive A-to-I RNA editing in limbic regions, generating enzyme variants with reduced catalytic activity; increased editing has been reported in amygdala samples from suicide victims and individuals with substance-use disorders (Grohmann et al., 2010), indicating that 5-HT synthetic capacity itself can be tuned at the RNA level. However, functional studies of TPH2 editing under stress and across sexes remain limited, despite evidence that TPH2 expression contributes to sex-linked differences in mood and anxiety phenotypes (Chen and Miller, 2012; 2013).

Among 5-HT receptor subtypes, the 5-HT_2C_ provides the most compelling example of editing-dependent functional diversification (Giorgetti and Tecott, 2004). Human and rodent 5-HT_2C_ receptor pre-mRNA undergoes A-to-I editing at up to five sites, generating receptor isoforms with graded reductions in constitutive activity, agonist potency, and G-protein-coupling efficiency (Burns et al., 1997; Niswender et al., 2001). Editing of *Htr2c* is dynamically reshaped by stress and antidepressant exposure. In high-anxiety BALB/c mice, early-life maternal separation or acute stress shifts forebrain *Htr2c* pre-mRNA toward more heavily edited, signaling-deficient isoforms, a change associated with increased depression-like behavior and reversible by chronic fluoxetine in a strain- and developmental-window–dependent manner (Englander et al., 2005; Bhansali et al., 2007; Mombereau et al., 2010).

In post-traumatic stress disorder-like paradigms, editing patterns within the central amygdala segregate resilient from susceptible animals and remain functionally reversible by acute 5-HT_2C_ antagonism (Warhaftig et al., 2021). Human post-mortem studies further indicate that cortical 5-HT_2C_ receptor editing abnormalities are more tightly linked to suicide than to MDD *per se*, with region-specific increases in editing associated with receptor variants exhibiting reduced G-protein coupling and, in some cases, decreased ADAR2 expression (Niswender et al., 2001; Lyddon et al., 2013; Weissmann et al., 2016). Consistently, knock-in mouse lines in which the five editable codons in *Htr2c* are genomically fixed to encode either non-edited or fully edited receptors display opposing anxiety- and antidepressant-like phenotypes and differential responses to desipramine, providing one of the clearest causal demonstrations that an RNA-editing pattern can shape serotonergic signaling and mood-related behavior *in vivo* (Mombereau et al., 2010). Collectively, these indicate how editing-dependent diversification of serotonergic components—particularly the 5-HT_2C_ receptor—can generate durable shifts in signaling landscapes and emotional behavior, even though the editing process itself remains dynamically regulated. However, most studies have relied on mixed-sex cohorts without sex-stratified molecular analyses, leaving unresolved whether RNA editing contributes to the well-documented sex differences in mood-disorder vulnerability. This gap highlights a critical area for future investigation within developmental programming models of serotonergic function.

### 7.4 m^6^A epitranscriptomic landscape: implications for serotonergic signaling

Beyond RNA editing, the brain epitranscriptome is also shaped by reversible RNA methylation, with N6-methyladenosine (m^6^A) representing the most prevalent internal modification in eukaryotic mRNAs which is dynamically regulated by writer/eraser/reader proteins, enabling rapid control of RNA fate (stability, translation, and localization) in processes tightly coupled to neuronal activity and stress responses (Ngo et al., 2025; Wang et al., 2025), position m^6^A as a plausible mechanism to understand how transient environmental signals may bias protein output during sensitive windows of circuit maturation.

Given the central role of stress-responsive plasticity in the pathophysiology of mood and stress-related psychiatric disorders, recent syntheses in psychiatric epitranscriptomics have begun to link alterations in m^6^A machinery and transcript-specific methylation profiles to depression-associated phenotypes across human post-mortem datasets and animal models, particularly within corticolimbic circuits (Ngo et al., 2025; Wang et al., 2025). In this context, human post-mortem prefrontal cortex from individuals with MDD, genome-wide m^6^A profiling reveals coordinated remodeling of methylation across transcripts enriched in synaptic and neuronal projection pathways (Mitsuhashi et al., 2024; Roy and Dwivedi, 2025). Notably, differential methylation signatures display sex-dependent features, with largely non-overlapping sets of altered transcripts in males and females. Nevertheless, these datasets remain correlative and do not clarify whether m^6^A alterations directly shape serotonergic dysfunction or reflect broader stress-related circuit pathology.

Complementary evidence from acute stress paradigms in male mice demonstrates rapid, region-specific m^6^A remodeling in limbic and prefrontal regions, and genetic manipulation of m^6^A regulators in adult hippocampal neurons alters fear-related learning and stress responsiveness (Engel et al., 2018). These findings indicate that m^6^A dynamics are acutely stress-sensitive and can modulate plasticity-relevant gene networks at the circuit level. Although serotonin-related transcripts were not among the differentially methylated targets identified under these acute conditions, both 5-HT receptor and SERT mRNAs harbor basal m^6^A/m peaks in cortical tissue, indicating that serotonergic mRNAs are part of the basal m^6^A epitranscriptome (Engel et al., 2018). Whether stress-induced m^6^A remodeling directly reshapes serotonergic signaling, particularly across development or in a sex-dependent manner, remains unresolved, as existing studies have been restricted to acute paradigms in male animals.

Direct experimental evidence linking m^6^A remodeling to serotonin-related transcripts remains limited but increasingly specific. In this context, studies in rodents demonstrate that m^6^A-dependent mechanisms can directly regulate defined 5-HT receptor mRNAs in neuronal systems: ALKBH5-mediated demethylation has been shown to stabilize 5-HT_3A_ and 5-HT_1B_ receptor mRNAs in distinct neuropathological contexts, altering receptor expression and neuronal excitability (Wu X. et al., 2023; Huang et al., 2024). In addition, components of the m^6^A machinery, such as ZC3H13, influence 5-HT release through regulation of ERICH3 in hippocampal neurons (Zhu et al., 2022), while manipulation of the eraser FTO in adult ventral tegmental area neurons reverses stress-induced depressive-like behaviors and normalizes expression of a subset of serotonergic transcripts, such as 5-HT_1D_ receptor (Wu et al., 2021). Although these findings indicate that serotonergic components are embedded within the m^6^A-regulated transcriptome, the extent to which m^6^A directly configures serotonergic tone during development or stress adaptation remains unresolved.

Together, these observations do not establish m^6^A as a primary driver of serotonergic dysfunction; rather, they position RNA modification as a mechanistically plausible layer through which stress-responsive and developmentally sensitive circuits, including 5-HT networks, may undergo lasting yet potentially reversible molecular reconfiguration. Notably, the enzymes that install and interpret these marks are themselves responsive to neuronal activity, hormonal signaling, and environmental stressors, suggesting that RNA-level regulation may constitute a point of convergence where sex-specific endocrine contexts intersect with serotonergic function. Within this framework, sex differences may emerge not solely through changes in transcript abundance, but also through subtle biases in RNA modification patterns that shape receptor signaling capacity, protein output, or circuit responsiveness. Although direct evidence remains limited, this perspective identifies RNA modifications as a tractable and high-priority mechanistic framework for future studies of sex-biased vulnerability to neurodevelopmental and affective disorders.

## 8 Concluding remarks

Taken together, the available evidence supports 5-HT as a timing-dependent organizer of brain development and a context-sensitive regulator of mature circuit function, whose perturbation can bias long-term neurobehavioral outcomes in a sex-dependent manner. Across developmental and adult models, the impact of altered serotonergic signaling is shaped not only by the direction of the perturbation but also by when it occurs, which circuits are engaged, and the endocrine and environmental context in which it unfolds. Yet a major unresolved challenge is to determine how early serotonergic disturbances are converted into persistent vulnerability to neurodevelopmental and, mainly, mood-related psychopathology, rather than adaptive or state-dependent reconfiguration alone. In this regard, sex should not be treated as a descriptive variable but rather as a biological dimension that may influence how serotonergic signals are integrated at the molecular, cellular, and circuit levels. RNA-centered regulatory mechanisms, including post-transcriptional control and epitranscriptome landscape, offer a plausible framework through which transient developmental or environmental insults may acquire lasting functional consequences without requiring parallel changes in transcript abundance alone. Defining how these regulatory layers intersect with developmental timing, circuit identity, and biological sex will be essential to advancing the field from correlative phenotypes to mechanistic models with genuine translational relevance.
